# Investigation on the Microstructure and Mechanical Properties of FeGa_3_ Surface Film on SKD11 Substrate

**DOI:** 10.3390/ma18184427

**Published:** 2025-09-22

**Authors:** Roonie Protasius, Masaki Tanaka, Shigeto Yamasaki, Tatsuya Morikawa, Kazuyuki Yagi, Masahiko Tezuka, Yasufumi Yoshida, Yukinari Yoshida, Makoto Higashionna

**Affiliations:** 1 Department of Materials, Kyushu University, 744 Motooka, Nishi-ku, Fukuoka-shi 819-0395, Japan; protasius.roonie.286@s.kyushu-u.ac.jp (R.P.); yamasaki.shigeto.259@m.kyushu-u.ac.jp (S.Y.); morikawa.tatsuya.771@m.kyushu-u.ac.jp (T.M.); 2 Department of Mechanical Engineering, Kyushu University, 744 Motooka, Nishi-ku, Fukuoka-shi 819-0395, Japan; yagik@mech.kyushu-u.ac.jp; 3 Next LM Development Department, X-Ray Tube Engineering Division, Canon Electron Tubes & Devices Co., Ltd., 1385, Shimoishigami, Otawara-shi 324-8550, Japan; masahiko.teduka@etd.canon (M.T.); yasufumi.yoshida@etd.canon (Y.Y.); yukinari.yoshida@etd.canon (Y.Y.); makoto1.higashionna@etd.canon (M.H.)

**Keywords:** surface film, FeGa_3_, SKD11, micro-cantilever, mechanical properties, microstructure

## Abstract

Gallium-based liquid metal is corrosive to steel alloys, forming FeGa_3_ surface films which can potentially be applied as a solid lubricant to enhance wear resistance and mitigate liquid metal-induced corrosion. However, the characteristics of these films remain insufficiently explored. In this study, Ga-In-Sn alloy was ultrasonically soldered onto annealed and decarburised substrates, followed by heating in a vacuum chamber to form a 30 μm thick FeGa_3_ reaction layer. The film on the annealed samples with an alpha-ferrite microstructure exhibited high porosity and a surface roughness of 1.97 Ra. In contrast, the film on the decarburised samples with a ferritic microstructure showed minimal porosity and a lower surface roughness of 1.29 Ra. Nanoindentation tests revealed Young modulus values of 231 GPa and 242 GPa and hardness values of 11.4 GPa and 12.7 GPa for the annealed and decarburised samples, respectively. The high porosity in the annealed samples is attributed to the suppression of FeGa_3_ formation in regions containing chromium carbides. Shear stress for fracture, measured by microcantilever tests at the interface between the substrate and the inner matrix of the surface film, showed lower fracture shear stress in the annealed sample, attributed to the presence of larger pores within its microstructure.

## 1. Introduction

Gallium-based liquid metals (GBLMs), which consist of more than 60 mass% gallium, are gaining attention due to their unique combination of properties. These include remarkably low melting points as low as −19 °C, low toxicity, excellent electrical/thermal conductivity, high-temperature stability, and effective lubricity, even under demanding conditions. Consequently, GBLMs find applications in diverse fields such as flexible electronics, biomedical devices, advanced sensors, nuclear reactor cooling, and aerospace thermal management [1,2,3]. While variants like Galinstan (Ga-In-Sn) have shown promise as lubricants in extreme environments [4], their interaction with common structural materials like steel presents challenges. GBLM can be corrosive, directly alloying with the substrate and leading to the formation of an intermetallic surface film, predominantly FeGa_3_, on steel surfaces [5,6]. This FeGa_3_ compound, possessing a tetragonal P42/mnm crystal structure, exhibits intriguing characteristics such as high hardness and a high melting point [2,7]. Notably, FeGa_3_ film bonds strongly to the substrate [2], potentially offers self-lubricating properties under dry sliding conditions [8], and, importantly, has been reported to inhibit further liquid metal (LM) corrosion on the steel substrate [5].

Prior works indicated that high chromium content, as found in stainless steel, can enhance resistance to LM corrosion [9]. This observation draws attention to other high-chromium steel alloys, such as SKD11 tool steel, which contains over 12 mass%. Cr and features a microstructure reinforced by platelet chromium carbides for strength [10]. While Cr-C films have shown GBLM resistance [11], and the base SKD11 composition suggests potential compatibility, no recorded studies have investigated the specific interaction between SKD11 and GBLM or characterised the resulting surface film. Furthermore, understanding the influence of microstructural features, particularly the presence or absence of large chromium carbides, on the formation of FeGa_3_ surface films has not been systematically investigated.

Understanding the nature of the FeGa_3_ surface film, such as morphology, composition, and thickness, formed on different substrates, is critical if it is intended to be used as an LM corrosion protection barrier. Geddis et al. [12] observed good corrosion resistance for SS304L and SS316L to GBLM up to 200 °C; however, it was reported that thick and porous compound layers consisting of FeGa_3_ and CrGa_4_ were formed when these stainless steel alloys were exposed to GBLM at 300–400 °C. Shin et al. [13] proposed a model of liquid metal corrosion on austenitic stainless steel, which highlighted the influence of the substrate on the formation of the surface film. These findings underscore the sensitivity of the reaction layer’s microstructure to processing parameters and substrate chemistry. Factors inherent to SKD11, such as its high chromium content and the presence of distinct chromium carbides, could potentially influence kinetics and the development of microstructural features like porosity, even under different thermal conditions than those of previous studies. The formation of such porosity is a critical concern, as it can significantly degrade mechanical properties such as hardness and elastic modulus [14] and moreover promote pathway for reaction between the corrosive medium and the substrate [15]. While the formation of FeGa_3_ on certain steels is documented, the detailed microstructure and mechanical behaviour of such a film on high-Cr, carbide-containing steels like SKD11 remain ambiguous.

For any surface film intended for wear protection or functional use, mechanical properties such as hardness (H), Young modulus (E), Poisson’s ratio (*v*), shear strength, and fracture behaviour dictate its performance [16,17,18]. Desirable characteristic often includes a high H/E surface film ratio and the value of the surface film’s Young modulus closely matching the substrates to ensure mechanical compatibility and durability. Guo et al. [2] examined FeGa_3_ surface film formed on T91 steel using Galinstan, reporting an average layer thickness of 26.8 μm with irregular, loose grains less than 5 μm in size. The nanoindentation hardness (H) and reduced modulus (E_r_) of the film were measured as 7.63 GPa and 241 GPa, respectively, close to the substrate modulus of 275 GPa. However, the inherent brittleness of the FeGa_3_ intermetallic compound, attributed to insufficient slip systems [19], poses challenges for wear application. Buckley and Johnson [8] highlighted the importance of shear strength and film adhesion to the substrate during sliding, as excessive shearing can exacerbate substrate reaction.

Accurate mechanical characterisation of thin, brittle films like FeGa_3_ present significant challenges due to the small length scale involved and the potential influence of the underlaying substrate [20,21], especially when microstructural defects like porosity are present. Scratch testing is commonly used to asses adhesion of film to the substrate [2,22], while nanoindentation tests are widely adopted for evaluating the hardness and Young’s modulus of thin films due to their localised nature and relatively simple sample preparation [23]. Depending on the type of surface film, a nanoindentation test may not be enough to understand its mechanical properties due to the fact that the mechanical responses under compressive and tensile stresses are different, especially for brittle surface films [24]. One of the methods that has been utilised to measure the mechanical response for tensile conditions is performed with a micro-sized cantilever fabricated by using a focus ion beam (FIB) [25,26,27]. The free end of the microcantilever beam is loaded using a nanoindenter, where the load displacement curve is obtained [20,24]. For example, Yamaguchi et al. successfully applied this method to characterise a thin, brittle BaTiO_3_ film [24].

In the present study, we apply this combined methodology to evaluate the microstructure and mechanical properties of the surface film formed on SKD11 with and without large chromium carbide precipitates. The influence of porosity and chromium carbides on the mechanical response of bulk FeGa_3_ under shear stress was also investigated.

## 2. Materials and Methods

**Figure 1:**
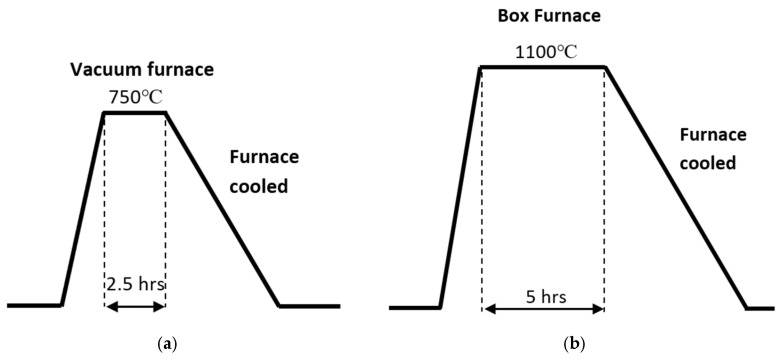
Schematic diagrams of the heat treatments, showing (**a**) the annealing parameters for Sample A and (**b**) the decarburisation parameters for Sample B.

**Figure 2:**
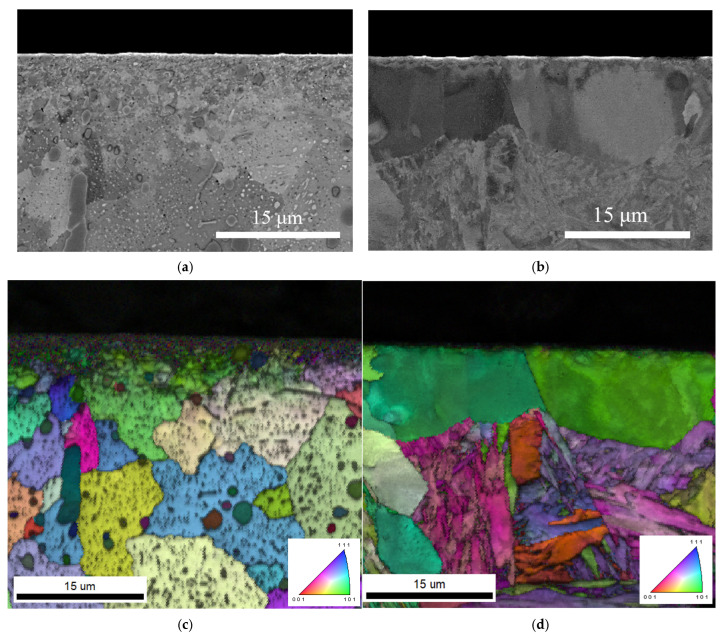
(**a**,**b**) BSE images showing the microstructure of Sample A and B and (**c**,**d**) IQ-IPF map showing the grain distribution in SKD11 matrix before the film formation.

An alloy tool steel of SKD11, commercially available, was used for the substrate. The chemical composition from the Japan Industrial Standard is shown in Table 1. Two types of substrates were prepared: an annealed SKD11 (Sample A) and a surface-decarburised SKD11 (Sample B). Heat diagrams are shown in Figure 1a and Figure 1b for Sample A and Sample B, respectively. Sample B was prepared to distinguish the effect of dissolving large chromium carbide precipitates in the SKD11 substrate on the formation of the FeGa_3_ compound. The decarburisation method was selected based on the successful dissolution of chromium carbides in air at 1000 °C, as reported by Takahashi et al. [28]. The oxide scale, which was produced during the decarburisation process, was removed. The sample surfaces were then mirror-polished to 0.04 ≥ R_a_ then ultrasonically cleaned in acetone and ethanol and dried in air. Figure 2 shows backscattered diffraction images and image quality–inverse pole figure (IQ-IPF) maps, showing the microstructure of Samples A and B before the compounds were formed. Liquid Ga-In-Sn was then ultrasonically soldered (Sunbonder USM-560, Komura-Tech, Higashiosaka, Osaka, Japan) on both surfaces of Samples A and B according to the method by Auger et al. [29] and then heated up in a vacuum chamber at 400 °C for 5 h to form FeGa_3_ reaction layers with a thickness of 30 μm. A total of 20 samples were prepared, with 10 samples for Sample A and the remaining 10 for Sample B. After cleaning in an ultrasonic bath, the surface roughness of the surface films formed was measured using a stylus profilometer (Surtronic S-100, Taylor Hobson, Leicester, UK) in accordance with ISO 4287 [30] and 4288 [31] standards.

The phase composition on the FeGa_3_ surface for both samples was identified using X-ray diffraction (XRD, Smartlab, Rigaku Corporation, Akatsuki, Osaka, Japan), using Cu-Kα radiation and an angle of 15 to 80° with a scan speed of 0.6°/min at 0.01° intervals. This was followed by analysis of the diffraction peak using PDXL2 Version 2.9.1.0 analysis software. For microstructural analysis, 3 randomly selected samples from the pool of Samples A and B were first cross-sectioned, mounted on conductive resin, and polished, involving the use of emery paper with a grit size ranging from #320 to #3000, followed by polishing with diamond slurry (grain size: 1 μm and 0.1 μm) and finally using 0.25 μm colloidal silica. Microstructural observations were conducted using a field-emission scanning electron microscope (FE-SEM, SU5000, HITACHI, Gurgaon, India) with an accelerating voltage of 15 kV. The element mapping on the sample’s cross-section was conducted using energy-dispersive spectroscopy (EDS, HITS4300SE, EDAX, Mahwah, NJ, USA) with an accelerating voltage of 25 kV. Electron backscatter diffraction (EBSD) analysis was employed in addition to the FE–SEM at an accelerating voltage of 25 kV and spot size of 50. Definition files for the FeGa_3_ compound were constructed and simulated using OIM Analysis 8.0 and open source ReciPro software [33], based on the detected phase during XRD analysis, and data from the material project website [34] and crystallographic open database (COD).

**Figure 3:**
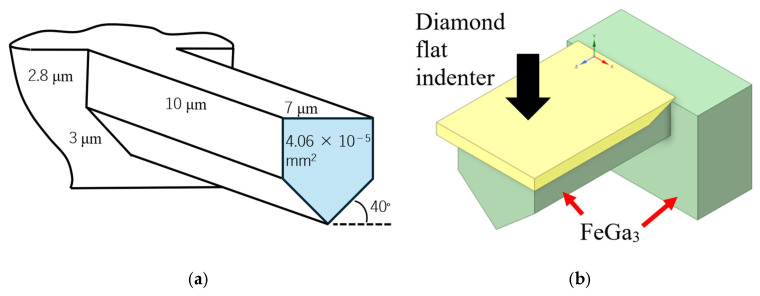
(**a**) Schematic drawing of the fabricated micro-sized cantilever beam for Samples A and B using focus ion beam. (**b**) Schematic illustration of the test set up for the shearing test using a flat punch diamond indenter on the free end of the microcantilever beam for both samples, where the blue arrow indicates the direction of the indenter movement.

Microcantilever beam samples were fabricated using the focus ion beam technique (FIB-SEM, Quanta 3D 200i, ThermoFisher Scientific, Waltham, MA, USA) on the cross-section of the FeGa_3_ layer of both samples. For each sample, three microcantilever beams were prepared at the interface between the respective surface film and the substrate, and another three were fabricated at the centre of the surface film. An acceleration voltage of 30 kV and current of 15 nA were used to produce the microcantilever with the dimensions shown in Figure 3a.

Nanoindentation tests were carried out (ENT-1100, Elionix, Tokyo, Japan) to investigate the hardness and elastic modulus of the FeGa_3_ layer as well as the substrate with a load from 0.25 mN to 10 mN for the FeGa_3_ layer and 5 mN for the substrate, respectively. A minimum of 20 indentation tests were performed using each indentation load across the cross-sectioned samples. Each line from the top surface to the substrate contained at least 10 indentation points. Shear tests on the microcantilever beam were conducted using a nanoindenter (ENT-5, Elionix, Tokyo, Japan) with a flat-punch indentation tip, where the whole cantilever beam’s surface was pressed, with a displacement speed of 15 nm/s, the schematic of which is shown in Figure 3b. The shear stress at the notch root was estimated using Equation (1), where *τ* is the shear stress, F is the load at failure, and A is the cross-sectional area of the microcantilever beam [24].

(1) $$\tau =\frac{F}{A}$$

The porosity of the FeGa_3_ surface film in both Samples A and B was measured using digital image analysis (DIA) on the SEM images, following the method by Grove and Jerram [35], with the JPOR macro in the open-code programme ImageJ 1.54P [36]. The porous area was taken as a percentage from the total area for each measured location, where the average percentage were then calculated. At least five areas were taken for each sample on the FeGa_3_ surface film.

## 3. Results

### 3.1. Microstructure

**Figure 4:**
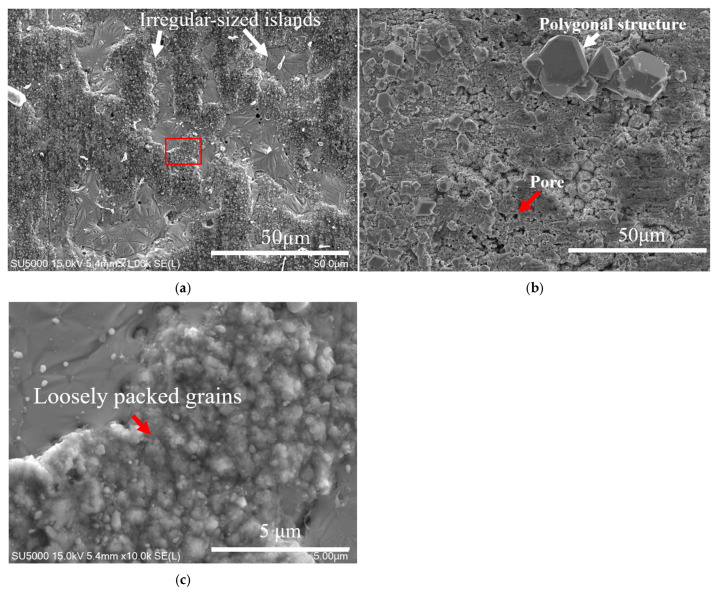
Enlarged SEM images showing the top surface of (**a**) Sample A and (**b**) Sample B after heat treatment. (**c**) The enlarged SEM image from the red box in (**a**).

Figure 4a,b show SEM images of the top surfaces of Samples A and B after the heat treatment, respectively. Figure 4a reveals that Sample A exhibits a surface primarily composed of irregular, loosely packed grains, as indicated by the red arrow in the enlarged SEM image in Figure 4c. Scattered across this granular matrix are distinct, smooth islands of irregular size and shape, highlighted by the white arrows in Figure 4a. In contrast, Figure 4b shows that Sample B also exhibits a matrix of irregular, loosely packed grains; however, it also shows that small pores are distributed across the surface, as well as scattered polygonal structures of various sizes, as indicated by the respective red and white arrows in the figure. Quantitative surface roughness measurement using a stylus profilometer showed a value of 1.97 R_a_ and 1.29 R_a_ for Sample A and Sample B, respectively, indicating a smoother surface topography for Sample B at the micrometre scale.

**Figure 5:**
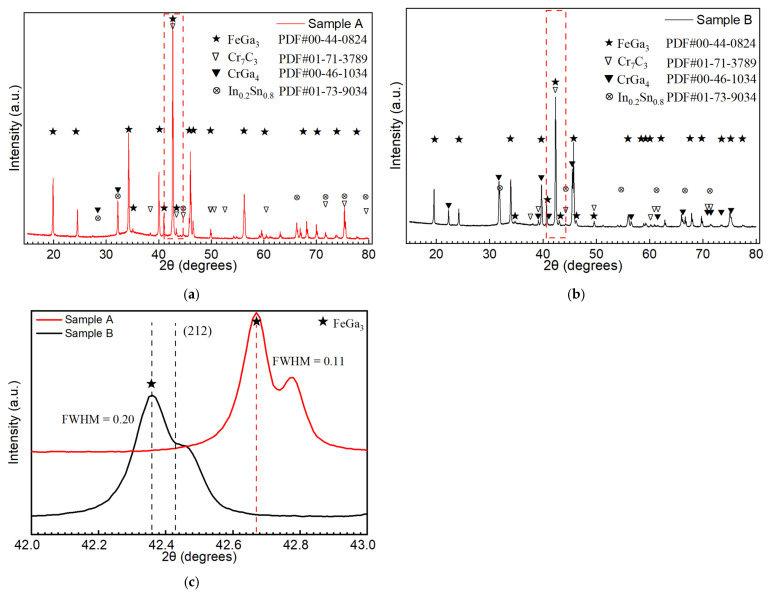
Phase identification of (**a**) Sample A and (**b**) Sample B, showing the XRD pattern. (**c**) Enlarged XRD pattern of the red dashed box area in Figure 5a,b. The dashed vertical line in (**c**) indicate the peak positions of Sample B and Sample A, which correspond to the (212) plane of the FeGa_3_ reference pattern.

Figure 5a,b show XRD profiles taken from the top surface of Samples A and B. They show matching peaks with the standard card #00-44-0824, indicating that FeGa_3_ is the dominant phase formed by the reaction of Fe and liquid Ga-In-Sn. Figure 5c shows the enlarged area surrounded by a red rectangle in Figure 5a,b, which shows the highest intensity peak for FeGa_3_ located at (2 1 2). Peak broadening is seen in both samples, which gives the full-width half-maximum (FWHM) value of 0.11 and 0.20 for Sample A and B, respectively. This broadening suggests the presence of nanocrystalline or sub-micron grains within the FeGa_3_ phase for both samples. According to the Scherrer relationship [37], the smaller FWHM of 0.11 for Sample A suggest potentially larger average crystallite sizes compared to Sample B with an FWHM of 0.20 [38]. Furthermore, this broadening is presumed to be influenced by the presence of residual strain in the FeGa_3_ grain for both samples. Depending on whether the residual stress is compressive or tensile, this will have a significant effect on the potential application of the surface film [39].

Figure 5a also shows the peaks associated with chromium compound labelled by the triangle symbol, matching those in the standard PDF card #01-71-3789 and #00-46-1034, indicating the presence of the Cr_7_C_3_ and CrGa_4_ compounds in the surface film. It is to be stressed that In-Sn compounds are detected at major peaks of the PDF card #01-073-9034 for Sample A; however, they are less detected for Sample B, marked by the circular symbol in Figure 5a,b. This suggests substantial segregation and solidification of a distinct In-Sn phase in Sample A, whereas in Sample B, these elements are presumed to be incorporated into the FeGa_3_ lattice, to exist below the XRD detection limit, or to be segregated in a different manner.

**Figure 6:**
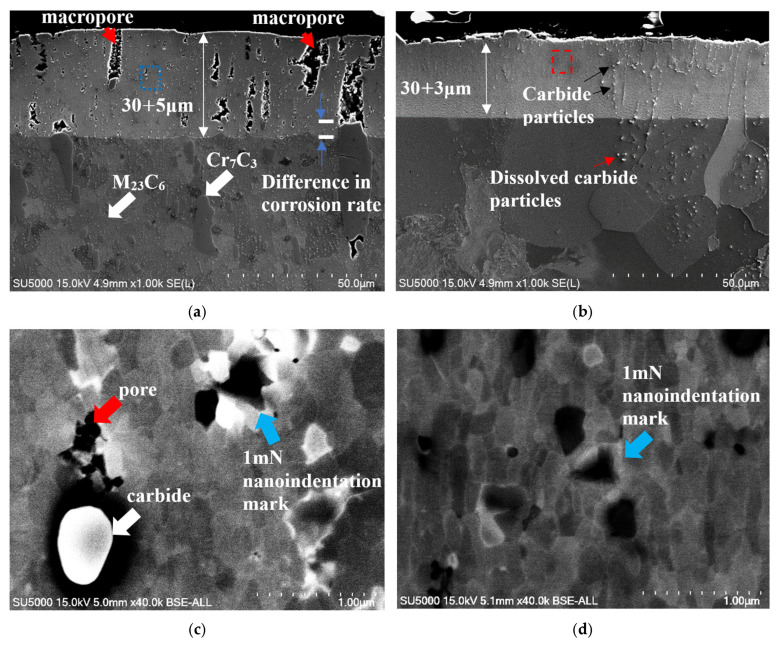

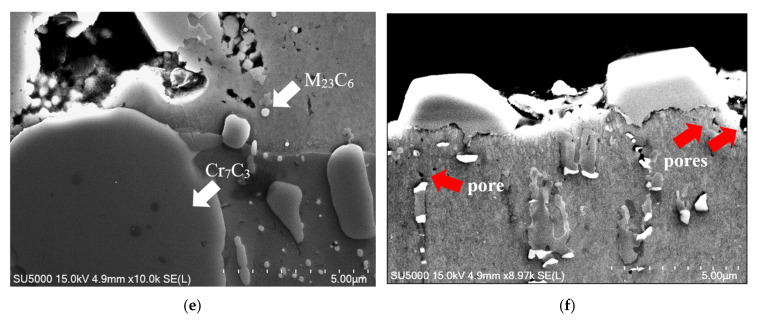
SEM images showing the cross-sectional surface after the heat treatments. (**a**,**b**) Cross-sectional morphology of Samples A and B. (**c**,**d**) Enlarged BSE images taken from the blue and red box area in (**a**,**b**), showing the presence of carbide particle and voids in Sample A and the grain size for both samples, as well as the indent size at 1 mN load. (**e**) Enlarged SEM image, showing the interface between the FeGa_3_ surface film and the SKD11 substrate. (**f**) Enlarged SEM image of the top surface of Sample B, showing the polygonal-shaped structure.

Figure 6a,b show SEM images of the cross-sections near the top surfaces of Samples A and B, respectively. Figure 6a shows that the FeGa_3_ surface film on Sample A has a thickness of 30 + 5 μm with a high number of macropores, indicated by the red arrows. The substantial internal porosity throughout the layer thickness is estimated to be 8.4 ± 5.2% by image binarization. Figure 6c shows an enlarged image from the area indicated by the blue rectangle in Figure 6a, where fine chromium carbides are embedded in the FeGa_3_ layer (indicated by the white arrow in Figure 6c), which originated from the SKD11 substrate, as seen in the SEM image of the interface between the FeGa_3_ layer and the SKD11 substrate in Figure 6e. The pores, indicated by the red arrow, formed in the vicinity of chromium carbide as shown in Figure 6c. The carbides also lead to an inhomogeneous corrosion rate, which results in a rough interface between the surface film and substrate, as indicated by the difference in depth between the highest peak and lowest depth indicated by the blue arrows in Figure 6a, where the difference can be seen at the position of large carbide particles in the SKD11 matrix. Large-sized Cr_7_C_3_ and smaller-sized M_23_C_6_ particles have been distributed in the matrix of SKD11 [10], as indicated by the white arrows in Figure 6a,e.

Figure 6b shows the cross-section of Sample B, indicating significantly fewer internal pores and a much denser FeGa_3_ layer with a thickness of 30 + 3 μm. The distribution of pores is not uniform. The porosities, as indicated by the red arrows in Figure 6f, 3 μm beneath the top surface, are estimated to be 10.8 ± 1.8% by image binarization. From this depth onwards, toward the interface of the substrate, the porosity is estimated to be 0.2 ± 0.1%. Throughout the layer, the average porosity is estimated to be 1.14 ± 0.2%. A small number and size of carbide particles are embedded in the FeGa_3_ layer, as indicated by the black arrows in Figure 6b. Consistent with XRD findings, polygonal-shaped crystals considered CrGa_4_ are detected at peaks of 31.77°, 39.26°, 39.76°, and 45.72° in the XRD profile shown in Figure 5b with the standard card #00-046-1034 and are observed on the outer surface of the FeGa_3_ layer; an enlarged image of the crystal structure here can be seen in Figure 6f.

**Figure 7:**
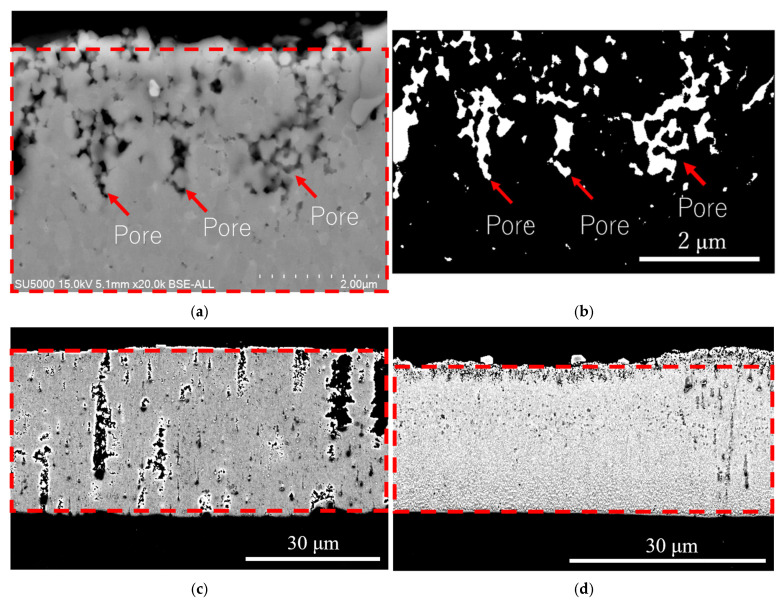
(**a**) BSE image taken 3 μm from the top surface of the cross-section of Sample B. The area in the red box in (**a**) is then binarized, and the result is shown in (**b**). (**c**) The BSE image of the cross-section of Sample A. (**d**) The BSE image of the cross-section of Sample B.

Figure 7 shows the image binarization process to estimate the porosity of Samples A and B. A BSE image is first acquired at the location where the porosity estimation is required, as shown in Figure 7a. This image corresponds to a region located 3 μm below the top surface of the cross-section of Sample B. The area containing pores, indicated by the red arrows in Figure 7a, is then selected using ImageJ software. This selected area is binarized using the JPOR macro, with the identified pores highlighted by the red arrows in Figure 7b. The porosity is subsequently calculated automatically by the software. A similar procedure is applied to estimate the porosity of both Samples A and B. Areas indicated by the red boxes in Figure 7c,d are first selected; these areas correspond to the cross-section of Samples A and B, respectively. The selected areas are then binarized, and the porosity values are obtained.

As a result of Cr_7_C_3_ extinction due to decarburization, a flat interface between the FeGa_3_ layer and the substrate SKD11 was obtained for Sample B, as shown in Figure 6b. Meanwhile, the decarburised SKD11 substrate has a larger grain size as a result of the decarburisation process compared to the substrate of Sample A. In addition to this, large-sized chromium carbides had dissolved in the substrate near the interface.

**Figure 8:**
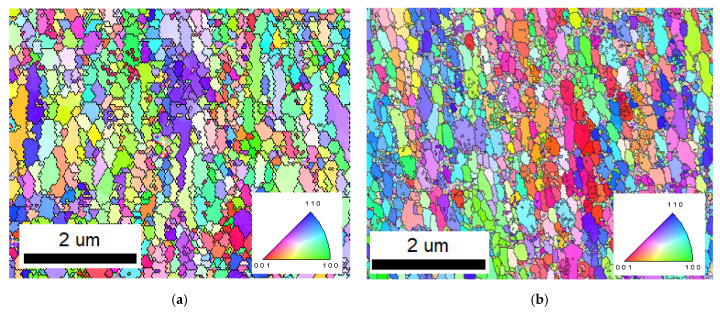
IPF maps taken from the centre of the surface film, showing the grain size distribution for (**a**) Sample A and (**b**) Sample B.

The FeGa_3_ matrices in both Samples A and B consist of irregularly shaped, sub-micron grains with an approximate circle average diameter (CAD) of 0.2 μm, as shown by the IPF map in Figure 8a,b. Given that FeGa_3_ is the primary composition of the surface film, these grains are reasonably assumed to be FeGa_3_. Contrary to previous finding [2], the grain size of the FeGa_3_ appears significantly smaller. However, this observation aligns with reports of nano-sized FeGa_3_ grains in other studies in the literature [4].

**Figure 9:**
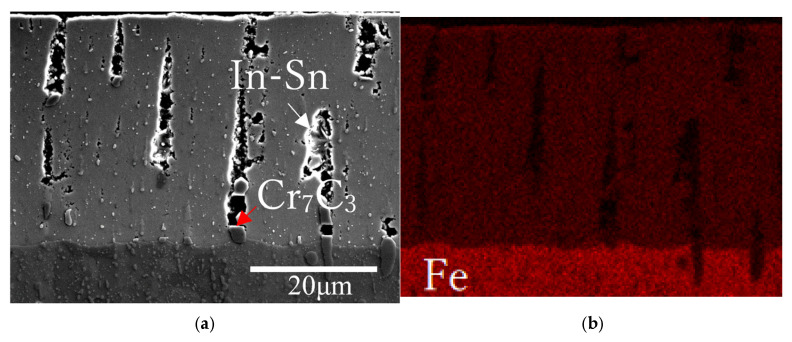

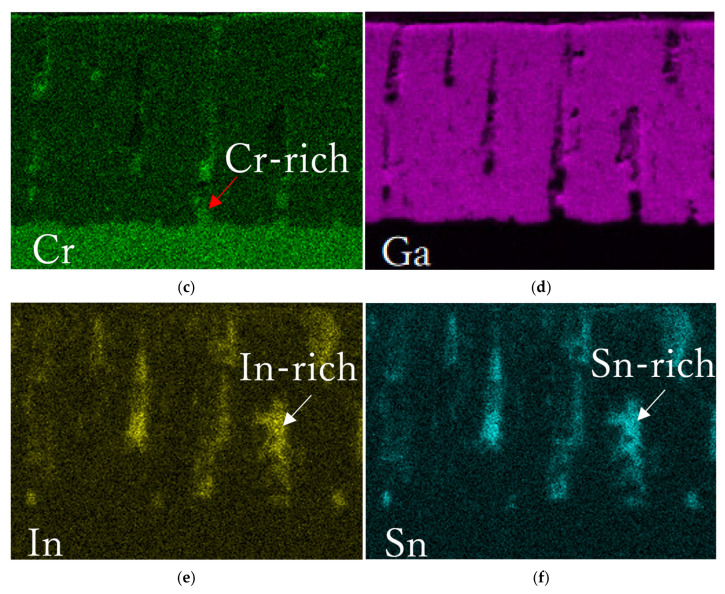
(**a**) SEM image and (**b**–**f**) EDS maps of the cross-sectional chemical composition in the reaction layer for Sample A.

Figure 9 shows EDS maps of the cross-section of Sample A, indicating the existence of both Fe and Ga in the FeGa_3_ layer, as the layer is mainly composed of the FeGa_3_ compound. A high concentration of Cr was detected in several location, such as the one shown by the red arrow in Figure 9c, correlating with the detection of chromium carbide in the FeGa_3_ compound for Sample A determined using XRD. It is presumed that pores near the area where Cr concentrates indicate the location where chromium carbide had existed, as indicated by the red arrow in Figure 9a. Notably, In and Sn signals in Figure 9e and Figure 9f, respectively, are also concentrated in these Cr-rich regions, often adjacent to pores, as shown in Figure 9a. This co-localisation suggests that the observed porosity is associated with the prior presence of the In-Sn phase, which preferentially segregates near the embedded chromium carbides. Features resembling the smooth island in Figure 4a are also observed within the cross-section, rich in In and Sn, as indicated by the white arrows in Figure 9a,e,f.

**Figure 10:**
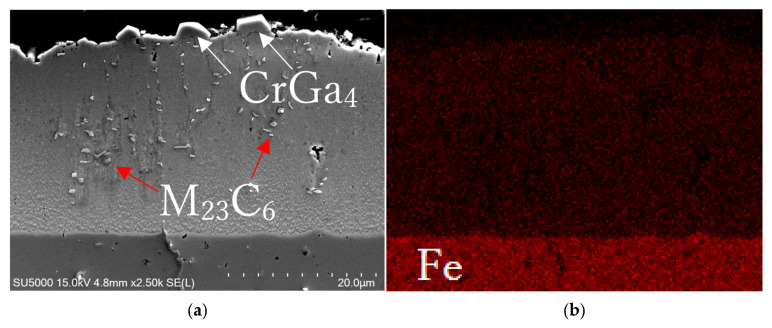

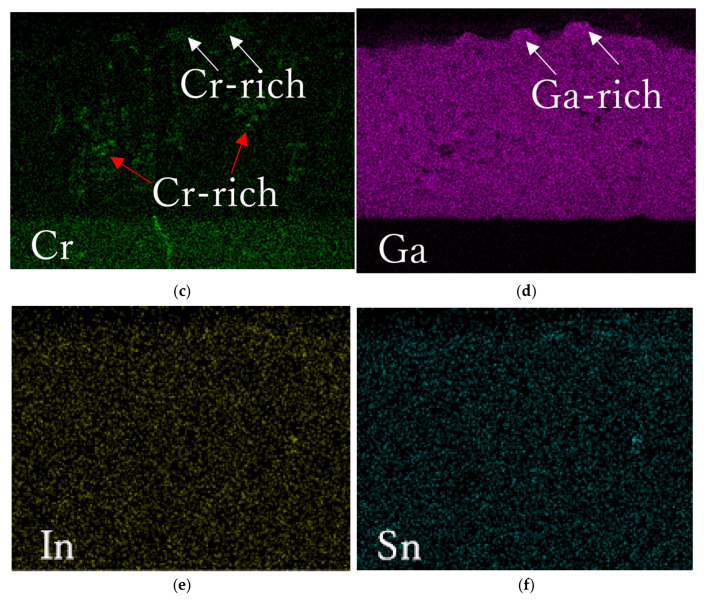
(**a**) SEM image and (**b**–**f**) EDS maps of the cross-sectional chemical composition in the reaction layer of Sample B.

Figure 10 shows EDS maps on the cross-section of Sample B, showing similar detection for Fe and Ga, as shown in Figure 9, which is attributed to the FeGa_3_ compound. The small particles are conceded to be M_23_C_6_ due to the size and the high concentration of Cr, as indicated by the red arrows in Figure 10a,c. The high concentration of Cr and Ga near the surface, as indicated by the white arrows in Figure 10a,c,d, corresponds to the precipitates shown in Figure 6f. It is considered to be CrGa_4_, as reported by Yu et al. [40] when stainless steels were corroded with gallium-based liquid metals at high temperatures.

### 3.2. Mechanical Test

**Figure 11:**
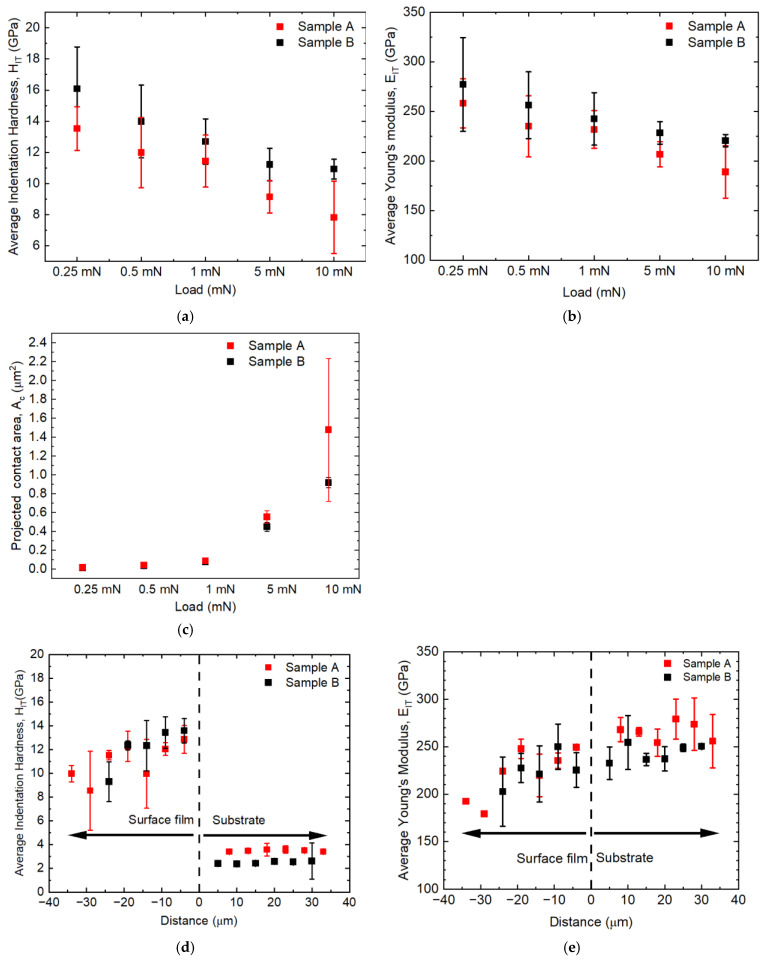
Some mechanical properties obtained from nanoindentation tests for Samples A and B. (**a**) Average hardness with different indentation loads. (**b**) Young modulus with different indentation loads. (**c**) The projected indentation area at different indentation loads, based on the function of indentation depth. (**d**) Hardness across the surface film towards the substrate. (**e**) Young modulus across the surface film towards the substrate. Nanoindentation tests on the cross-section of the compound layer were conducted using a load of 1 mN, while those for the substrate were conducted using a load of 5 mN.

In order to reveal the mechanical properties of FeGa_3_, a series of nanoindentation tests were conducted. These tests aimed to determine the hardness obtained by nanoindentation measurement and Young’s modulus as a function of the applied load, investigate the property across the film–substrate interface, and estimate the effective bulk mechanical properties by accounting for film porosity. Figure 11a shows the average hardness (H_IT_) for Samples A and B, measured at the centre of their surface films. H_IT_ generally decreases as the load increases from 0.25 mN to 10 mN in both samples. Sample A yielded an average H_IT_ of 13.5 ± 1.4 GPa at 0.25 mN, appeared to stabilise around 11.4 ± 1.7 GPa at 1 mN, and subsequently decreased to 7.8 ± 2.3 GPa at 10 mN. Sample B demonstrated a comparable pattern, although the results suggest that H_IT_ could be higher compared to Sample A. Its H_IT_ was 16.1 ± 2.7 GPa at 0.25 mN, decreased to 12.7 ± 1.5 GPa at 1 mN, and then appeared to reach a more stable value of approximately 11 GPa at 5 mN and 10 mN.

Figure 11b presents the average Young modulus (E_IT_) for Samples A and B. Similarly to H_IT_, E_IT_ decreased as the load increased from 0.25 mN to 10 mN. For Sample A, E_IT_ was at 258 ± 25 GPa at 0.25 mN and decreased to 232 ± 19 GPa at 1 mN, and it further decreased to 189 ± 27 GPa at a 10 mN load. The average values for Sample B are generally higher compared to Sample A across all loads. The E_IT_ value for Sample B was 277.1 ± 47.3 GPa at 0.25 mN, dropping to 242 ± 27 GPa at a 1 mN load and stabilizing to 220 ±6 GPa at 10 mN.

The large standard deviation, especially at lower loads, should reflect the influence of the local microstructure such as the crystallographic orientation, number of grains involved, and grain boundaries [41]. To verify the above, the projected contact area was firstly calculated (A_c_). The commonly used equation in nanoindentation of H = P/A_c_ was used to obtain the A_c_, where H is the indentation hardness obtained from the nanoindentation test and P is the applied load [42]. The calculated A_c_ based on applied load and indentation hardness is shown in Figure 11c. At an indentation load of 1 mN, the A_c_ was approximately 0.087 μm^2^ for Sample A and 0.081 μm^2^ for Sample B. Based on the relationship between indentation depth (h_c_) and projected contact area, A_c_ = 24.504 h_c_^2^ [23], this yield h_c_ would be approximately 59 nm for Sample A and 57 nm for Sample B. The characteristic lateral dimension of a Berkovich indent is approximately 7.5 x h_c_ [23]. Thus, at 1 mN, the indent size is approximately 0.44 μm in lateral side length. Comparing this indent dimension with the average grain size of Samples A and B, it is evident that indentation at 1 mN interacts with a few grains, as indicated by the indent mark denoted by the blue arrows in Figure 6c,d. As the load further increased beyond 1 mN, A_c_ exponentially increased, as depicted in Figure 11c. This is due to cracking from the nanoindents, where the value of H_IT_ does not show the actual value. Consequently, a 1 mN load was selected as the reference load for further indentation tests across the surface films.

The values of H_IT_ were measured across the cross-section. Loads of 1 mN and 5 mN were applied to the compound layer and the substrate, respectively. Figure 11d shows the average value of H_IT_, indicating that H_IT_ varies across the surface layer while it is nearly constant in the matrix. The lowest hardness inside the compound layer of Sample A was recorded near the top surface of 8.5 ± 3.3 GPa, while the hardness at the centre of the compound layer is 11 ± 2 GPa. While the cross-section of the surface film has been properly polished to reduce measurement error [43], the greater spread of values in Sample A may suggest material heterogeneity across the surface film [44]. Overall, the H_IT_ of the compound is more than three times higher than that of the substrate of 3 ± 0.8 GPa. Meanwhile, the compound layer of Sample B also shows lower H_IT_ at the top surface of 9 ± 1.7 GPa but a more consistent reading at the centre of the compound of 13 ± 1.5 GPa. The H_IT_ on the substrate is 2 ± 0.13 GPa. The H_IT_ of the compound is approximately six times higher than that of the substrate.

Figure 11e shows E_IT_ across the cross-section, the value of which is taken simultaneously with H_IT_, showing the same trend with E_IT_. The lowest value for Sample A was found to be near the top surface of 179 ± 0.13 GPa, while the value fluctuates toward the centre, with an average of 232 ± 19 GPa. The average value of E_IT_ for the substrate was 266 ± 19 GPa, which is slightly higher than that for the compound layer. The E_IT_ of the compound layer for Sample B is more consistent across the compound layer and close to the value for the substrate. The average values for E_IT_ for the compound layer and the substrate are 242 ± 27 GPa and 243 ± 15 GPa, respectively, although a slight reduction in E_IT_ can be observed at the top surface of the surface film of 203 ± 37 GPa.

**Figure 12:**
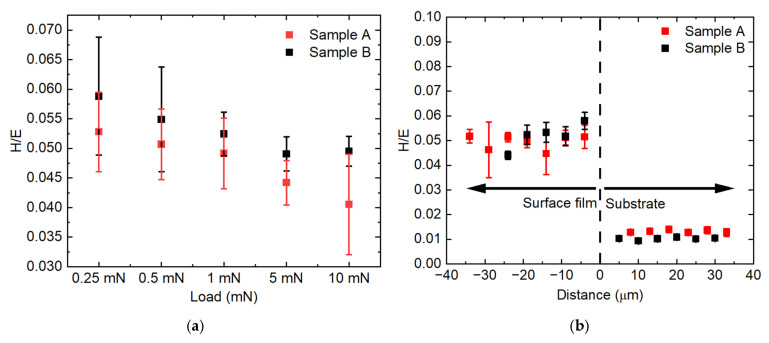
(**a**) The plasticity index (H/E ratio) of Sample A and B plotted against the applied load. (**b**) The H/E ratio of Sample A and B plotted across the surface film towards the substrate.

Figure 12a shows the hardness over Young modulus (H/E) ratio, plotted against the nanoindentation load. The value of H/E for Sample A was 0.053 ± 0.007 at 0.25 mN, decreased to 0.049 ± 0.005 at 1 m N, and further decreased to 0.041 ± 0.009 at a 10 mN load. The H/E value for Sample B was 0.059 ± 0.010 at 0.25 mN, dropping to 0.052 ± 0.004 at 1 mN and stabilizing to 0.050 ± 0.002 at 10 mN. Overall, Sample B indicated a higher value for H/E compared to Sample A at all indentation loads but showed a much more stable value at higher indentation loads.

As explained in the previous section, 1 mN for the compound layer and 5 mN for the substrate are used to plot the H/E ratio for Samples A and B across the surface film towards the substrate, as shown in Figure 11b. The H/E values for both samples remain nearly constant at 0.5 across the surface film. In contrast, the H/E values for both substrates are consistently lower, around 0.01. The higher H/E ratios observed in the compound layers of Sample A and B, compared to their respective substrate, may indicate superior wear resistance of the compound layers [45,46].

To validate the E_IT_ value obtained from the nanoindentation test, the E_IT_ was compared with that retrieved from the Materials Project for Ga_3_Fe (mp-636368) from database version v2023.11.1. Here, the value of the Poisson ratio, *v*, and the bulk modulus, K, for FeGa_3_ were reported to be 0.23 and 101 GPa, respectively [34]. The Young modulus obtained with the equation of E = 3K(1 − 2*v*) [47] was estimated to be 168 GPa. The reference value has good agreement with results from Sample A at a higher load but is significantly lower compared to results from Sample B at all loads. Additionally, the inconsistency in the H_IT_ and E_IT_ value, especially for Sample A, prompted further investigation.

### 3.3. Microcantilever Test

**Figure 13:**
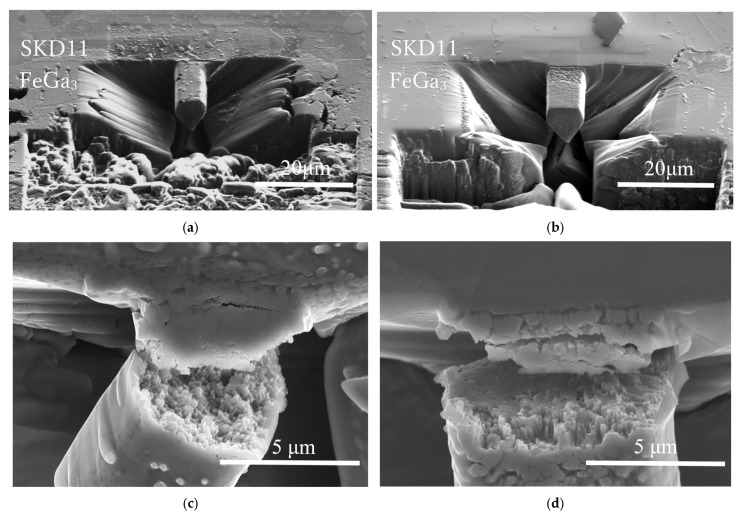
SEM images of the fabricated cantilever at the interface of (**a**) Sample A and (**b**) Sample B before the shearing test. SEM images showing the fractured cantilever for (**c**) Sample A and (**d**) Sample B after the shearing test at the interface.

Figure 13a,b show fabricated microcantilever beams for Samples A and B, respectively, formed at the interface between the FeGa_3_ compound layer and the substrate before the shearing tests. White contrasts on the surface in Sample A indicate the presence of chromium carbide precipitates embedded in the cantilever beam, while Sample B has a smoother surface, indicating a lower number of chromium carbide precipitates in the cantilever beam. Figure 13c,d show fracture surfaces of the microcantilever after shearing tests. The fracture surface of Sample A is rough, showing intergranular failure and failure through a region with microstructural heterogeneity. On the other hand, the fracture surface of Sample B, as shown in Figure 13d, is smoother with a small number of macropores.

**Figure 14:**
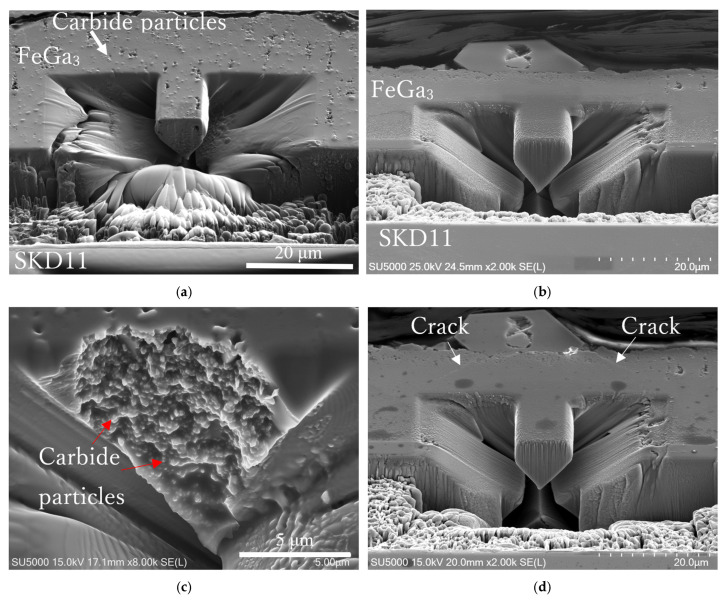
(**a**,**b**) SEM images of the fabricated cantilever before the shearing test for Sample A and Sample B, respectively. (**c**) Enlarged image of the fractured surface of the cantilever in Sample A. (**d**) SEM image of the fractured, porous top surface of Sample B.

Figure 14a,b show fabricated microcantilever beams inside the compound layer for Samples A and B before the shearing tests, respectively. The cantilevers were fabricated at a minimum distance of 10 μm from the top surface of both samples. The fracture surface of Sample A in Figure 14c shows the existence of macropores and individual particle-like features, indicating the high porosity of the internal structure of the microcantilever beam, as well as the existence of chromium carbide particles (red arrows) in the inner matrix of Sample A. Fracture has likely propagated through the interconnected pores and along the weakly bonded chromium carbide particle interfaces. On the other hand, the microcantilever beam of Sample B itself did not fracture, although the crack propagated from the porous top surface, as shown in Figure 14d. This indicates that the compound itself in Sample B is much more resistant to fracture compared to that in Sample A.

**Figure 15:**
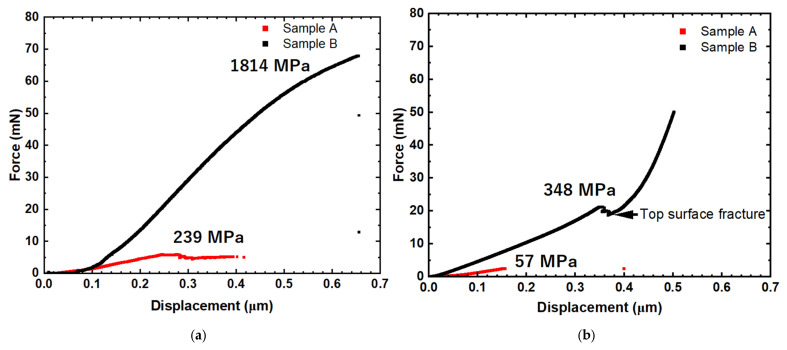
Load–displacement curves for the shear test on Samples A and B. The cantilever was formed (**a**) at the interface between the substrate and the respective FeGa_3_ and (**b**) at the centre of the FeGa_3_ compound layer.

Figure 15a shows the load–displacement curves obtained from bending tests with the cantilever at the interface. The red curve is for Sample A, exhibiting a maximum load of around 6 mN at a displacement of 0.26 μm, corresponding to a calculated shear stress of 239 MPa, where the cross-sectional area of the cantilever is 2.4 × 10^−5^ mm^2^. The black curve is for Sample B, showing a much steeper initial slope, indicating higher stiffness compared to Sample A. The force increases almost linearly to much higher loads, leading to fracture at a load of 68 mN. The shear stress at fracture is expected to be approximately 1814 MPa with the cross-section of the cantilever of 3.7 × 10^−5^ mm^2^. The shear stress for the fracture of Sample B is approximately 7.5 times higher than that of Sample A. It is presumed that Sample B, with low porosity, shows the true strength of the compound layer.

Figure 15b shows the force-displacement curves obtained from the cantilevers formed inside the compound layer. The red curve is from Sample A, indicating that Sample A fractured at the load of 2.4 mN, which corresponds to 57 MPa. The black curve is from Sample B, indicating that the microcantilever beam for Sample B itself did not fracture completely; instead, cracks were initiated from the top surface, as indicated by the white arrows in Figure 14d. This initiation of cracks led to the sudden drop in load indicated by the black arrow in Figure 15b. Further application of shearing loads to the microcantilever until reaching 50 mN also did not fracture the cantilever. These results indicate that the compound layer in Sample B has higher resistance to fracture than Sample A.

## 4. Discussion

### 4.1. Microstructure and Mechanical Properties

It was indicated that the overall porosity of the FeGa_3_ surface film in Sample A is higher and distributed throughout the compound layer. In contrast, the porosity of Sample B is concentrated at the top surface and homogenous inside. This is because GBLM has low reactivity and does not readily wet chromium carbide with higher chromium content [11]. The chromium carbide of SKD11 mainly consists of Cr_7_C_3_ and M_23_C_6_ with a chromium-to-carbon ratio of 7:3 and 8:2, respectively. Previous work by Lindersson [11] shows that GBLM does react with chromium carbide film with a lower chromium content with a Cr-to-C ratio of 3.7:6.3. At the same time, the solubility of Fe and Cr in liquid Ga at 400 °C follows the order of Fe > Cr [9]. Therefore, while the GBLM alloy reacts first with the Fe in the annealed base material to form the FeGa_3_ layer, the Cr-rich carbide remains as it is. As the FeGa_3_ layer grows, the GBLM still does not readily wet the surface of the Cr-rich carbide in the SKD11 interface. This makes the GBLM react with the Fe surrounding the Cr-rich carbide. If another large particle of Cr-rich carbide is present, as indicated by the red arrow in Figure 9a, this can lead to multiple pores, and as more Cr-rich carbide particles are present, a high-porosity layer is produced.

The decarburisation process of Sample B was successful in dissolving the large primary chromium carbides and small secondary chromium carbides, which were originally present in the base material. The dissolution of these Cr-rich carbides dispersed the Cr element farly evenly throughout the decarburised interface, as shown in Figure 9c. This resulted in the formation of more uniform FeGa_3_ at the interface between the substrate of Sample B and the compound layer, preventing the formation of large voids, as seen in Sample A. It is to be noted that exposure of the gallium-based liquid metal to a high chromium ferrous alloy at high temperature is still subject to corrosion, as reported in other studies [12]. The observation of CrGa_4_ on the surface of Sample B aligns with such high-temperature reactions.

The differences in porosity and carbide distribution influence the mechanical properties. The compound layer in Sample A, with its higher porosity and embedded particles, generally shows a lower hardness, lower Young modulus, and significantly lower shear strength compared to Sample B. This suggests that the macroscopic elastic constant differed due to the existence of pores.

Guo et al. [2] measured the values of hardness and elastic modulus for FeGa_3_ film formed on T91 steel, showing H_IT_ and E_r_ values of 7.63 ± 0.31 GPa and 241.3 ± 1.8, respectively, using a 5 mN indentation load. The values are almost identical to the values for Sample A with the same load. However, their values are lower than those for Sample B, as indicated in Figure 11a,b. Although the porosity of their samples was not measured, and their SEM image of the grain structure resembles the loose grain of Sample A, it is presumed that the FeGa_3_ compound that they formed contained some porosity, which contributed to the inferior mechanical properties.

Ternero et al. have shown that a porosity of 10% results in a decrease of 30% in Young modulus [48]. The porosity of Sample A was approximately 8.4 ± 5.2% throughout the surface film, as mentioned in Figure 6a, which can be interpreted as a reduction in Young modulus due to the higher porosity. The smoother interface and denser compound layer at the centre of Sample B contributed to its superior mechanical performance, including better matching of its Young modulus with the substrate.

### 4.2. Microcantilever Beam Shearing Test

The microcantilever shear test revealed the influence of porosity on the actual strength of the bulk FeGa_3_ compound. The porosities were caused by the excessive presence of chromium carbide particles, as discussed in the previous subsection. Although the values for the Young modulus for FeGa_3_ obtained by nanoindentation tests were not much different between Samples A and B, the slope of the load–displacement curve in Figure 15 is not identical. The change in slope corresponds to the change in shear modulus, as the simple shear tests were conducted using microcantilevers in this study. The difference in the slope indicates that the bulk shear modulus of Sample B is higher than that of Sample A. The slope is considered to be influenced mainly by the presence of inherent porosity. That is, the higher slope in Sample B is due to the low porosity of 0.2% where the cantilever was formed. This trend was also observed in shear tests conducted within the inner surface compound layer, where Sample A exhibited lower shear strength than Sample B. In Sample B, crack propagation initiated at the top surface due to porosity, causing it to fail under lower shear loads, while the central region remained intact.

The friction reduction [8] is attributed to the shearing of the FeGa_3_ surface film. As the surface film heats up, a thin lubricating film forms between the sliding surfaces. However, if the whole compound is sheared away, the protective layer is lost [5], exposing the substrate to further reaction with the liquid gallium. In the case of Sample A, despite the FeGa_3_ compound’s high hardness and Young modulus, the lower shear strength of the surface film makes it more susceptible to shearing during sliding. While this may enhance friction and wear reduction, it also increases the risk of substrate corrosion. In contrast, Sample B demonstrated higher shear strength at both the interface and the centre of the surface film, with only the top surface showing lower shear resistance. As the temperature rises, the sheared top surface contributes to friction and wear reduction, while the intact central region continues to protect the substrate from LM corrosion.

While the H/E index has been discussed, and the obtained values exhibit that both compound films indicated higher wear resistance compared to the substrate, the microcantilever shearing test indicated that the more porous microcantilever beam fractured at the lower shear stress. This suggests that the value of porosity needs to be taken into account to investigate the wear resistance of the material using this index, which means that the index gives a better indication of the wear resistance of porous materials, as well as true density materials. In addition, further wear tests, such as the disc sliding test, will be conducted on the compound layers to give a better indication of the effect of porosity on the shearing of the surface film.

It has been reported that higher initial surface roughness intensifies wear on the sliding surface due to the smaller contact area between the sliding pair, leading to higher contact pressure [49]. Although a longer running-in period for surfaces with higher Ra has been reported, it was also found that the peaks and valleys of such surfaces can retain lubricant more effectively during lubricated sliding, thus performing much better than smoother surfaces [50]. While there is a slight difference in Ra values between the two samples, and the Ra values are higher than those of conventional wear-protective coatings with high hardness such as TiAlN [51], it is expected that the wear rate of both samples will be relatively high during dry sliding. At the same time, the running in time or distance for Sample A could be longer than that for Sample B. However, if the surface films of Samples A and B are applied as an LM protective barrier by utilising LM as a lubricant, it is expected that the friction will be reduced tremendously [4] and the SKD11 substrate will be protected from further LM corrosion [5].

## 5. Conclusions

Mechanical properties of FeGa_3_ formed on SKD 11 were investigated. The following results were obtained:

(1) The FeGa_3_ surface film on the annealed SKD11 substrate exhibits porosity of up to 8% across the surface film, due to the inability of the reaction between the gallium-based liquid metal and chromium-rich carbide precipitates of Cr_7_C_3_ and M_23_C_6_. In contrast, the FeGa_3_ on the decarburised SKD11 substrate showed porosity below 1% across the surface film, due to the dissolution of these precipitates into the matrix.
(2) The intrinsic hardness of the FeGa_3_ compound on both substrates was consistently measured at approximately 11–13 GPa.
(3) The decarburisation process significantly enhanced the shear strength of the compound layer formed. The layer on the decarburised SKD11 substrate showed more than six times higher shear strength than that on the annealed substrate, indicating enhanced potential for practical applications under high mechanical stresses.

## Figures and Tables

**Table 1:** Chemical composition of SKD 11 (mass%) [32].

Material	C	Si	Mn	P	S	Cr	V	Fe
SKD11	1.4–1.6	0.4≥	0.6≥	0.03≥	0.03≥	11–13	0.2–0.5	Bal.

## Data Availability

The original contributions presented in this study are included in the article. Further inquiries can be directed to the corresponding author.
